# Comparison of Mating Disruption and Insecticide Application for Control of Peachtree Borer and Lesser Peachtree Borer (Lepidoptera: Sesiidae) in Peach

**DOI:** 10.3390/insects11100658

**Published:** 2020-09-25

**Authors:** Daniel L. Frank, Stephen Starcher, Rakesh S. Chandran

**Affiliations:** 1 Department of Entomology, College of Agriculture and Life Sciences, Virginia Tech, Blacksburg, VA 24061, USA; 2 Extension Service, Agriculture and Natural Resources Unit, West Virginia University, Morgantown, WV 26506, USA; RSChandran@mail.wvu.edu; 3 Division of Applied Sciences, Potomac State College, Keyser, WV 26726, USA; stephen.starcher@mail.wvu.edu

**Keywords:** Integrated Pest Management, behavioral control, pheromone, *Synanthedon exitiosa*, *Synanthedon pictipes*

## Abstract

**Simple Summary:**

The peachtree borer and lesser peachtree borer are destructive insect pests of peach and other stone fruit. Damage is caused by larval feeding on the vascular cambium, which reduces tree health and vigor and can lead to tree death. The traditional method to control these pests is with directed trunk sprays of the organophosphate insecticide, chlorpyrifos. However, the ongoing review of pesticide tolerances mandated by the United States Environmental Protection Agency (US-EPA) makes the option of future long-term use of chlorpyrifos uncertain. Mating disruption is an alternative method of control that uses synthetic sex pheromones to disrupt male mate-finding behavior and prevent mating. We conducted field studies to compare the efficacy of mating disruption and application of chlorpyrifos insecticide for control of this pest complex. Although our data indicated that mating disruption can provide growers with an effective non-chemical alternative to chlorpyrifos trunk sprays, several variables may affect its long-term success in West Virginia peach orchards; most notably the presence of high population densities of the pest species, problems with maintaining adequate pheromone coverage in orchards, and the need for area-wide implementation.

**Abstract:**

The peachtree borer, *Synanthedon exitiosa*, and lesser peachtree borer, *S. pictipes*, are economically important indirect pests of peach in West Virginia. The purpose of this 3-year study was to compare the efficacy of mating disruption and post-harvest trunk sprays of chlorpyrifos insecticide for control of this pest complex in a commercial peach orchard. Overall, Isomate PTB-Dual disruption dispensers applied at a rate of 371/ha significantly disrupted the male mate-finding behavior of *S. exitiosa* and *S. pictipes*. In addition, the infestation of peach trees by *S. exitiosa* larvae did not vary significantly between mating disruption and insecticide treated plots. Hot-spot maps of *S. exitiosa* infestation showed significant spatial clusters of infestation predominately near the perimeter of all orchard plots, or where trees were missing within and/or between rows. The generation of standard deviational ellipses revelated that the location of *S. exitiosa* infestations in orchard plots remained relatively constant between years, and were generally oriented in a north and easterly direction, which coincided with the prevailing wind direction. Although our data indicated that mating disruption can provide growers with an effective non-chemical alternative to chlorpyrifos trunk sprays, several variables may affect its long-term success in West Virginia peach orchards; most notably the presence of high population densities, problems with maintaining adequate pheromone coverage, and the need for area-wide implementation.

## 1. Introduction

Peachtree borer, *Synanthedon exitiosa* (Say) (Lepidoptera: Sesiidae), and lesser peachtree borer, *Synanthedon pictipes* Grote and Robinson (Lepidoptera: Sesiidae) are economically important wood-boring pests of peach, *Prunus persica* [L.] Batsch, and other cultivated stone fruit in eastern North America. Larvae of both species feed on the cambium tissue of host trees, disrupting the plant vascular system. Damage from *S. exitiosa* is generally confined to the trunk and roots near the soil line, while damage from *S. pictipes* occurs in the upper trunk and scaffold limbs. Consecutive seasons of infestation and larval feeding can result in the decline in tree health and vigor [1,2,3], increase susceptibility to disease and damage from other pests [1,3], or, in severe cases, cause tree death from girdling of major scaffold limbs and/or the trunk [1,4,5].

Behavioral manipulation of pest insects using sex pheromones is an environmentally benign tactic that has received increasing attention in pest control, and can be readily aligned with other management strategies [6]. Pheromone-based mating disruption has been used to disrupt the male mate-finding behavior of numerous Lepidopteran species [7], including several important sesiid pests [8,9,10,11,12]. The compound, (Z,Z)-3,13-octadecadien-1-ol acetate (ODDA), is the main component of the female sex pheromone of *S. exitiosa*, while (E,Z)-3,13 ODDA, in its pure form, is the female sex pheromone of *S. pictipes* [13]. Early attempts at *S. exitiosa* and *S. pictipes* mating disruption using a pheromone blend containing a 70:30 ratio of (E,Z):(Z,Z)-3,13 ODDA resulted in the elimination of male captures in traps, and provided varying degrees of success in reducing larval infestations in peach orchards [14]. Registered for commercial sale in 2010, Isomate PTB-Dual mating disruption dispensers were developed for control of both *S. exitiosa* and *S. pictipes*, and contain a 50:50 ratio of (E,Z):(Z,Z)-3,13 ODDA. Current guidelines for commercial peach growers recommend Isomate PTB-Dual for mating disruption in orchards where both borer species exist [15,16].

In West Virginia, peach orchards are non-contiguous and typically experience heavy pest pressure from both *S. exitiosa* and *S. pictipes*. Because studies with other lepidopteran species have shown that pheromone-based mating disruption is most effective when used area-wide and where low population densities of the target pest species exist [7,17], it remains uncertain if this pest management approach can provide the needed level of control to reduce *S. exitiosa* and *S. pictipes* population numbers in the primary peach-growing regions of West Virginia. Furthermore, the degree of control mating disruption can achieve in orchards relative to traditional chemical controls is unclear. Chemical control of *S. exitiosa* and *S. pictipes* currently centers on use of the organophosphate insecticide, chlorpyrifos to prevent larval entry into host trees [15,16]. The ongoing review of pesticide tolerances mandated by the United States Environmental Protection Agency (US-EPA) under the Food Quality Protection Act of 1996 led the agency to begin the process of cancelling registrations of the chemical in November 2016 [18]. However, this process was later reversed, which allowed the continuing legal use of chlorpyrifos pending future appeal. Increased restrictions on the use of chlorpyrifos in orchards and its uncertain future in the United States have emphasized the need for alternative management options for *S. exitiosa* and *S. pictipes*.

This study was conducted to determine whether the adoption of mating disruption could provide equivalent levels of *S. exitiosa* and *S. pictipes* control compared with traditional post-harvest trunk sprays of chlorpyrifos insecticide. Geospatial techniques were also used to investigate the distribution of *S. exitiosa* infestations in orchard plots under mating disruption and insecticide control programs.

## 2. Materials and Methods

### 2.1. Study Site and Treatments

Studies were conducted from 2015 to 2017 in a commercial peach orchard located in Hampshire County, WV. Six peach plots within the orchard were selected for the study and ranged in size from 0.6 to 1.9 ha. Each plot was planted with a mixture of peach cultivars with plot ages ranging from 5 to 8 years old. Peach cultivars included ‘Bounty’, ‘Contender’, ‘Gloria’, ‘PF-8 Ball’, ‘PF-28’, ‘Red Haven’, and ‘Summer Serenade’. Peach trees within plots measured ~3.0 m in height and were planted uniformly at a spacing of ~6.1 × 6.1 m. Although the orchard was under an active management program for arthropod pests and diseases during the study, none of the pesticide products applied as maintenance sprays would be expected to reduce infestations of *S. exitiosa* and *S. pictipes*.

Peach plots within the orchard were randomly assigned to either mating disruption or insecticide control treatments. Individual plots were spaced at least 100 m apart and bordered by woodlands or non-peach orchard farmland. In mating disruption treatments, Isomate PTB-Dual disruption dispensers (50:50 ratio of (E,Z):(Z,Z)-3,13 ODDA; CBC Amercia, Commack, NY, USA) were uniformly deployed within tree canopies at a height of ~2 m above the ground at a rate of 371/ha (150/A). Dispensers were applied in late April of each year before the emergence of moths. Insecticide treatments consisted of a handgun application of chlorpyrifos (2.8 L/378.5 L dilution) directed to the lower scaffold limbs and trunks of trees. Insecticide sprays were applied post-harvest during each year of the study.

### 2.2. Monitoring and Larval Infestation Assessments

Male moths were monitored using delta-style sticky traps (Suterra LLC, Bend, OR) baited with either a standard *S. exitiosa* or *S. pictipes* rubber septa pheromone lure (3146 and 3140, respectively; Trécé Inc., Adair, OK, USA). Three traps were deployed in each plot for each species. Traps were evenly spaced and placed in tree canopies at a height of ~1.8 m above the ground. All traps were checked at approximately weekly intervals throughout the adult flight period, and the number of male moths captured was recorded and moths removed.

Infestation of peach trees by *S. exitiosa* larvae was evaluated in May 2015 to assess infestation levels at the beginning of the study, and then again at the end of each season in November 2015, October 2016, and November 2017. Every tree was inspected within each plot and the number of active feeding sites were recorded. An active feeding site was considered to be a discrete and contiguous area of fresh frass and gummosis at the base of trees. Infestation of peach trees by *S. pictipes* was evaluated, similar to Pfeiffer et al. [19], where 10 groups of 10 trees were sampled from each plot for the presence of active feeding sites in cankers and pupal exuviae protruding from bark. Trees were examined twice in 2015 following the emergence of each generation. However, because few pupal exuviae were found during sampling, and the identification of active *S. pictipes* feeding sites was difficult to distinguish, only infestation by *S. exitiosa* is presented.

### 2.3. DataAnalysis

Capture of moths in traps for 2015, 2016, and 2017 were analyzed separately. A nonparametric Wilcoxon–Mann–Whitney test was performed (SAS 9.2, SAS Institute, Cary, NC, USA) to determine if there was a significant difference between the mean number of moths captured in pheromone-baited trap in the mating disruption and insecticide control plots. For larval infestation surveys, the number of *S. exitiosa* infested trees from each plot was converted to proportion infested by dividing the number infested by the total number of trees from each plot. The proportion data were analyzed using PROC GLIMMIX of the SAS statistical package. The model contained the main effect of treatment, sample date, and the interaction of treatment × sample date. Replication was included as the random variable while all other variables were fixed. Least squares means (LSMEANS) of fixed effects were calculated and comparisons were performed using the ADJUST = TUKEY option of the LSMEANS statement. Results from all tests were considered statistically different at *p* < 0.05.

A geographic information system (GIS) was used to provide a visual representation of the spatio-temporal distribution of *S. exitiosa* infestation in orchard plots. The location of trees and active *S. exitiosa* feeding sites were entered in a desktop GIS package (ArcGIS 10.7, ESRI Inc., Redlands, CA, USA) to locate and analyze the data. Using the “optimized hot spot analysis” tool in ArcGIS, hot-spot maps were created to identify statistically significant spatial clusters of infestation in orchard plots. The tool is based on the Getis-Ord Gi* statistic [20], which compares the sum of each spatial feature including its neighbors to the sum expected under the assumption of random spatial distribution of the values. Hot-spots were then assigned using statistically significant groups placed into 90, 95, and 99% confidence intervals. Weighted standard deviational ellipses (SDE) were also generated to visualize the spatial attributes (location, orientation, and dispersion) of infestation in orchard plots during each year of the study.

## 3. Results

### 3.1. Capture of Moths in Traps

**Figure 1:**
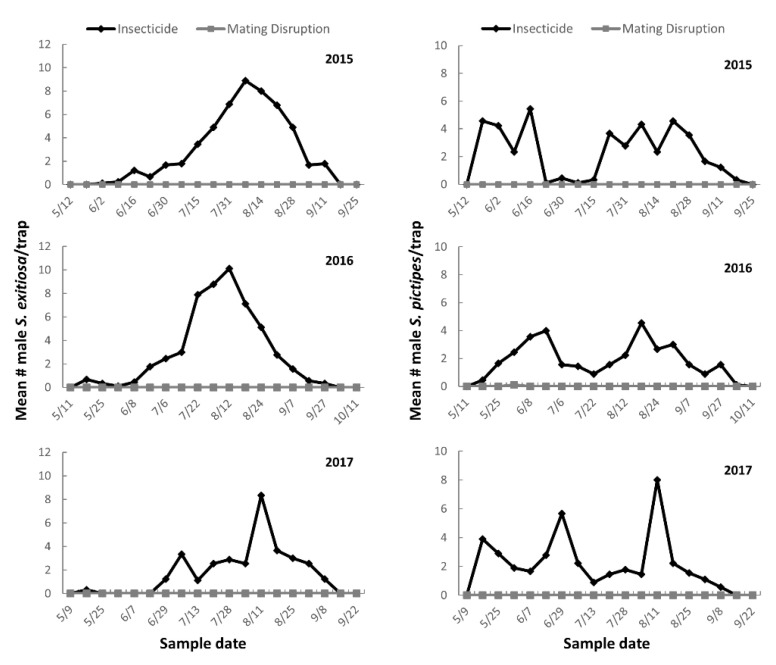
Mean number of male *S. exitiosa* and *S. pictipes* captured throughout the season in pheromone-baited traps in 2015–2017.

In West Virginia, *S. exitiosa* has a single generation/year while *S. pictipes* has two generations/year, which was evident by the respective unimodal and bimodal flight patterns of male moths in the insecticide treated plots (Figure 1). In the mating disruption plots, capture of male moths in pheromone-baited traps was virtually eliminated throughout the study period (Figure 1). Significantly more male *S. exitiosa* were captured in traps within insecticide treated plots compared with the mating disruption plots in 2015 (*z* = 3.77; *p* ≤ 0.0001), 2016 (*z* = 3.77; *p* ≤ 0.0001), and 2017 (*z* = 3.77; *p* ≤ 0.0001) (Table 1). Similarly, significantly more male *S. pictipes* were captured in traps within insecticide treated plots compared with the mating disruption plots in 2015 (*z* = 3.78; *p* ≤ 0.0001), 2016 (*z* = 3.77; *p* ≤ 0.0001), and 2017 (*z* = 3.78; *p* ≤ 0.0001) (Table 1).

### 3.2. Infestation

**Figure 2:**
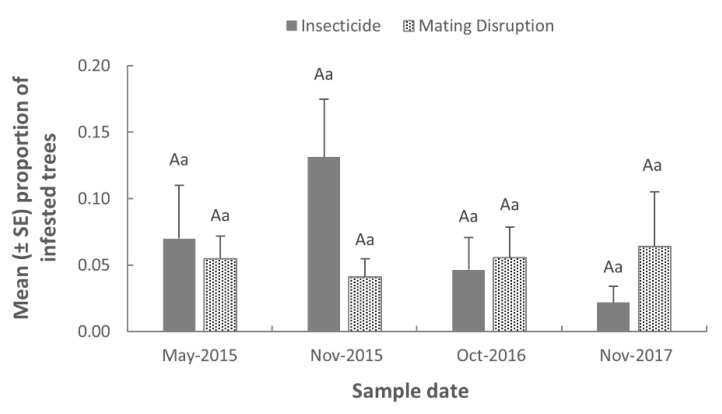
Mean (±SE) proportion of peach trees infested with *S. exitiosa* larvae in plots treated with insecticide or mating disruption. Uppercase letters indicate comparisons between treatments within each sample date (*p* > 0.05). Lowercase letters indicate comparisons among sample dates within each treatment (*p* > 0.05).

The proportion of infested trees in orchard plots did not vary significantly among treatment (*F* = 0.02; df = 1,15; *p* = 0.8848), sample date (*F* = 0.59; df = 3,15; *p* = 0.6295), and their interaction (*F* = 1.61; df = 3,15; *p* = 0.2285). When the effect of treatment was examined within each sample date, no significant difference in the proportion of infested trees was observed (Figure 2). Similarly, there was no significant difference in the proportion of infested trees among sample dates within each treatment (Figure 2).

### 3.3. Spatio-Temporal Distribution

**Figure 3:**
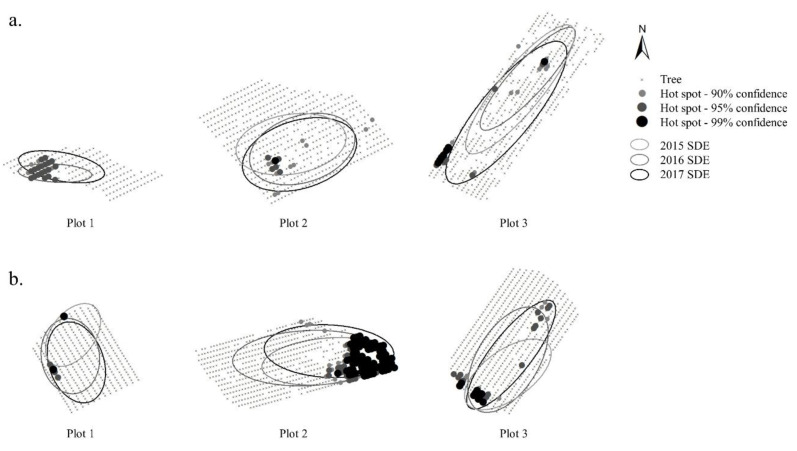
Getis-Ord Gi* optimized hot spot analysis of *S. exitiosa* infestation in plots treated with (**a**) insecticide and (**b**) mating disruption during the study period (2015–2017). Maps also show standard deviation ellipses (SDE) of the infestation area for each year.

Hot-spot maps of *S. exitiosa* infestation within orchard plots are shown in Figure 3. During the study, significant spatial clusters of infestation were observed predominately near the perimeter of plots, or where trees were missing within and/or between rows. The standard deviation ellipses (SDE) in Figure 3 display the spatial attributes of *S. exitiosa* infestation in plots during each year of the study. The location of infestations based on the SDE remained relatively constant between years. Infestations were generally oriented in a north and easterly direction, which coincided with the prevailing wind direction at the study site. The size of the SDE in plots differed between years. In insecticide treated plots the mean area of the SDE was 7453, 4905, and 7682 m in 2015, 2016, and 2017, respectively. In mating disruption plots, the mean area of the SDE was 6284, 7469, and 5179 m in 2015, 2016, and 2017, respectively.

## 4. Discussion

Few studies evaluating mating disruption as a control option for *S. exitiosa* and *S. pictipes* have been published in the refereed literature. Early attempts at mating disruption for control of *S. pictipes* in peach orchards showed that a 70:30 ratio of (E,Z):(Z,Z)-3,13 ODDA provided a 19–97% reduction in pupal exuviae counts in treated plots relative to insecticide controls [19]. Agnello and Kain [14] demonstrated that the 70:30 pheromone blend provided similar levels of *S. exitiosa* and *S. pictipes* control compared to directed trunk sprays of the pyrethroid insecticide esfenvalerate. Although comparisons to insecticide controls were not conducted, Grasswitz and Yao [21] showed that commercially available Isomate PTB-Dual dispensers (50:50 ratio of [E,Z]:[Z,Z]-3,13 ODDA) disrupted captures of male *S. exitiosa* in pheromone-baited traps, and reduced larval infestation in peach orchards when used at a rate of approximately 500 dispensers/ha (202 dispensers/A). In this study, the mate-finding behavior of male *S. exitiosa* and *S. pictipes* was successfully disrupted by deployment of Isomate PTB-Dual mating disruption dispensers at the minimum recommended application rate of 371 dispensers/ha (150 dispensers/A). Furthermore, infestation of peach trees by *S. exitiosa* larvae did not vary significantly between mating disruption and insecticide control plots.

In West Virginia, grower surveys have indicated that *S. exitiosa* and *S. pictipes* are the primary pests of concern in peach orchards, and that effective alternatives to traditional chemical controls are desired to mitigate the decline in tree health and productivity caused by this pest complex. Although a post-harvest application of chlorpyrifos has been the traditional method of control, this option was often cited by growers as being labor intensive and difficult to apply in a timely manner. Many West Virginia peach growers have diversified farm operations where the harvest of other crops can immediately follow the peach season, thus delaying the application of insecticide until after the majority of larvae have become established in trees. Prior to the start of this study, orchard plots were actively managed for *S. exitiosa* and *S. pictipes* using chlorpyrifos insecticide. Despite previous use of this management tactic, infestation data collected during the May 2015 sample date showed that, on average, 6.2% (range, 0–14%) of trees within plots were already infested with *S. exitiosa* larvae, likely because of sub-optimal insecticide application timing during the post-harvest period. While chlorpyrifos applications were conducted immediately following harvest in 2016 and 2017, they were not applied until 3 weeks post-harvest in 2015, which may account for the numerical increase in the proportion of infested trees observed in insecticide-treated plots during fall 2015. Although the application of mating disruption technology can similarly be labor intensive to apply, the deployment of pheromone dispensers occurs before adult flight in the spring, and can provide season-long control that will not interfere with other farm production practices.

Although mating disruption provided equivalent levels of control relative to chlorpyrifos insecticide, infestation data collected from mating disruption plots showed that, on average, 5.4% of trees were infested with *S. exitiosa* larvae over the three-year period. Numerous factors can influence the success of pheromone-based management strategies such as pest population density, orchard size, and degree of isolation from adjacent non-pheromone treated areas [7]. In general, pheromone-based management strategies are most effective where low population densities of the target species exist, and where large areas are under treatment to combat the effects of immigrating females [17,22,23]. Given that the orchard plots used in this study were located in a prominent peach-producing region of the state where area-wide disruption programs have not been implemented, and where relatively high population levels of both pest species exist, it is highly likely that immigration of gravid females from outside the study area contributed to the annual presence of infestations in orchard plots. Although this hypothesis was not tested in the present study, visualization and analyses of the spatio-temporal distribution of *S. exitiosa* infestations showed that hotspots were generally more prevalent near the perimeter of orchard plots, and infestation patterns generally exhibited a north and easterly orientation that coincided with the prevailing wind direction.

Inadequate pheromone coverage may be another reason for the annual presence of *S. exitiosa* infestations in mating disruption plots. The depletion of pheromone concentration along field edges has been documented in orchards treated with mating disruption, which can enhance the probability of infestation rates in these areas [24]. Because the ratio of edge trees to interior trees is higher in small orchards, pheromone depletion may account for the breakdown in control observed along plot edges. Other areas where high infestation levels were observed included the interior of plots where tree gaps were present within and/or between rows. The presence of geological features in the landscape resulted in plot areas with missing trees, which may have left areas of little or no pheromone coverage, where mate-finding could occur.

Although our data indicate that pheromone-based mating disruption can provide a suitable alternative to insecticide, the high material cost of mating disruption relative to conventional insecticides can be an impediment to adoption by growers [25,26]. In this study, we used the lowest recommended application rate of pheromone dispensers to reduce costs and make mating disruption more competitive with insecticide use. However, if the immigration of gravid females from outside treated orchards results in population buildup over time, or orchard characteristics prevent adequate pheromone coverage, mating disruption can fail to control the target pest [7]. Under these conditions, higher application rates of pheromone dispensers or supplementary use of insecticides would likely be needed to enhance control efficacy. However, these additional costs could limit the wider adoption of this technology for many growers if cheaper insecticide control options exist.

## 5. Conclusions

In the future, if the United States Environmental Protection Agency (US-EPA) moves to issue a cancellation order for the federal registration of cholorpyrifos-containing products, or further restricts its use in tree fruit, growers will be increasingly forced to rely on mating disruption for control of *S. exitiosa* and *S. pictipes* in peach and other stone fruit plantings. Although mating disruption can provide growers with an effective non-chemical alternative to chlorpyrifos trunk sprays, several variables may affect its long-term success in West Virginia peach orchards, most notably the presence of high population densities and problems with maintaining adequate pheromone coverage. Additional studies will be needed to understand how best to manage these limitations to ensure adequate control. Furthermore, the expansion of educational programs for growers will be needed to highlight both the strengths and weaknesses of pheromone-based mating disruption as well as its implementation. The facilitation of area-wide programs would also aid grower adoption and control efforts.

## Figures and Tables

**Table 1:** Total and mean (±SE) number of male *S. exitiosa* and *S. pictipes* captured in pheromone baited traps deployed in insecticide and mating disruption plots in 2015–2017.

Year	Treatment	Total Number of Male Moths Captured in Traps		Mean (±SE) Number of Male Moths/Trap/Week	
		*S. exitiosa*	*S. pictipes*	*S. exitiosa*	*S. pictipes*
2015	Insecticide	476	378	2.8 ± 0.9 *	2.2 ± 1.3 *
	Mating Disruption	0	0	0.0 ± 0.0	0.0 ± 0.0
					
2016	Insecticide	477	307	2.8 ± 0.1 *	1.8 ± 0.7 *
	Mating Disruption	0	1	0.0 ± 0.0	0.0 ± 0.0
					
2017	Insecticide	295	366	1.7 ± 0.6 *	2.1 ± 0.7 *
	Mating Disruption	0	0	0.0 ± 0.0	0.0 ± 0.0

* Significant difference between traps deployed in mating disruption and insecticide-treated plots based on Wilcoxon–Mann–Whitney test (*p* < 0.05).
